# Non-invasive removal of a misplaced and knotted guidewire during ultrasound-guided central venous catheter insertion in a hybrid operating room: a case report

**DOI:** 10.1186/s40981-024-00761-w

**Published:** 2024-12-21

**Authors:** Mizuho Matsushita, Yoshikazu Yamaguchi, Honoka Yamashita, Chiyori Yamauchi, Hajime Hayami, Joseph D. Tobias, Gaku Inagawa

**Affiliations:** 1 Department of Anesthesiology, Yokohama Municipal Citizen’s Hospital, Yokohama, Japan; 2 Department of Anesthesiology & Pain Medicine, Nationwide Children’s Hospital and The Ohio State University Wexner Medical Center, Columbus, OH USA

**Keywords:** Central venous catheter, Complications, Guidewire, Ultrasound-guidance, Knot

## Abstract

**Background:**

The standard of care for placement of a central venous catheter (CVC) includes a real-time ultrasound (US)-guided technique. We describe a rare case in which the guidewire penetrated the posterior wall of the vessel, forming a knot, which precluded simple removal. This occurred despite the procedure being performed under real-time US guidance. The guidewire was eventually removed under fluoroscopic guidance in a hybrid operation room.

**Case presentation:**

An 89-year-old male underwent the placement of a CVC in the left internal jugular vein. During the US-guided procedure, the guidewire penetrated the posterior wall of the vessel and formed a knot, which impeded simple removal. This was confirmed by radiologic imaging. Using a short sheath and a push–pull technique, the radiologist was able to untangle the knot to allow for catheter removal. The guidewire was safely removed without vascular injury.

**Conclusions:**

A very rare complication of guidewire knotting was observed despite the use of US-guidance during needle and wire placement. The use of US, computed tomography, and fluoroscopy were beneficial for diagnosis, while the hybrid operating room provided the optimal environment for the removal procedure.

**Supplementary Information:**

The online version contains supplementary material available at 10.1186/s40981-024-00761-w.

## Introduction

Ultrasound (US)-guide central venous catheter (CVC) placement is generally considered safer than conventional anatomic landmark techniques [1]. Therefore, US-guided CVC placement is strongly recommended in practice guidelines to reduce the incidence of mechanical complications [2]. However, real-time US guidance cannot completely prevent mechanical complications such as catheter malposition, pneumothorax, and arterial injury. The current clinical guidelines do not address the diagnosis and management of CVC-associated trauma or injury, with the exception of cases involving carotid arterial injury. Therefore, this case-report is useful for all of physicians who perform CVC placement [3]. We present a case of US-guided venipuncture in which the guidewire penetrated the posterior wall of a blood vessel, formed a knot, and was difficult to remove using conventional manual traction and removal. US and computed tomography (CT) imaging of the neck were useful in diagnosing guidewire migration and knot formation. Guidewire removal was performed in the hybrid operating room suite under fluoroscopic guidance.

## Case presentation

Presentation of this case report is in line with the guidelines of the Institutional Review Board of Yokohama Municipal Citizen’s Hospital (Yokohama, Japan). Written informed consent for publication was obtained from the patient’s family.

The patient was an 89-year-old male diagnosed with subarachnoid hemorrhage. Neurosurgical clipping of the site of hemorrhage was performed. On postoperative day 16, the patient’s body temperature increased significantly from 37.6 °C to 39.4 °C, accompanied by a rise in C-reactive protein (CRP) levels from 6.4 mg/dL to 16.4 mg/dL, raising concerns about catheter-related bloodstream infection (CRBSI) in the CVC previously placed in the right internal jugular vein (IJV). Due to difficulties in securing a peripheral venous line and the risk of hemodynamic instability requiring norepinephrine infusion, a new CVC was planned for insertion via the left internal jugular vein (IJV). The patient was placed in the Trendelenburg position with his head turned to the right. The patient was sedated during the CVC insertion, receiving 40 mg/h of propofol, 0.4 mcg/kg/h of dexmedetomidine, and 10 mcg/h of fentanyl. A PGY (post-graduate year)−2 anesthesiology resident performed the initial CVC insertion under the supervision of a PGY-6 anesthesia attending physician.

**Fig. 1:**
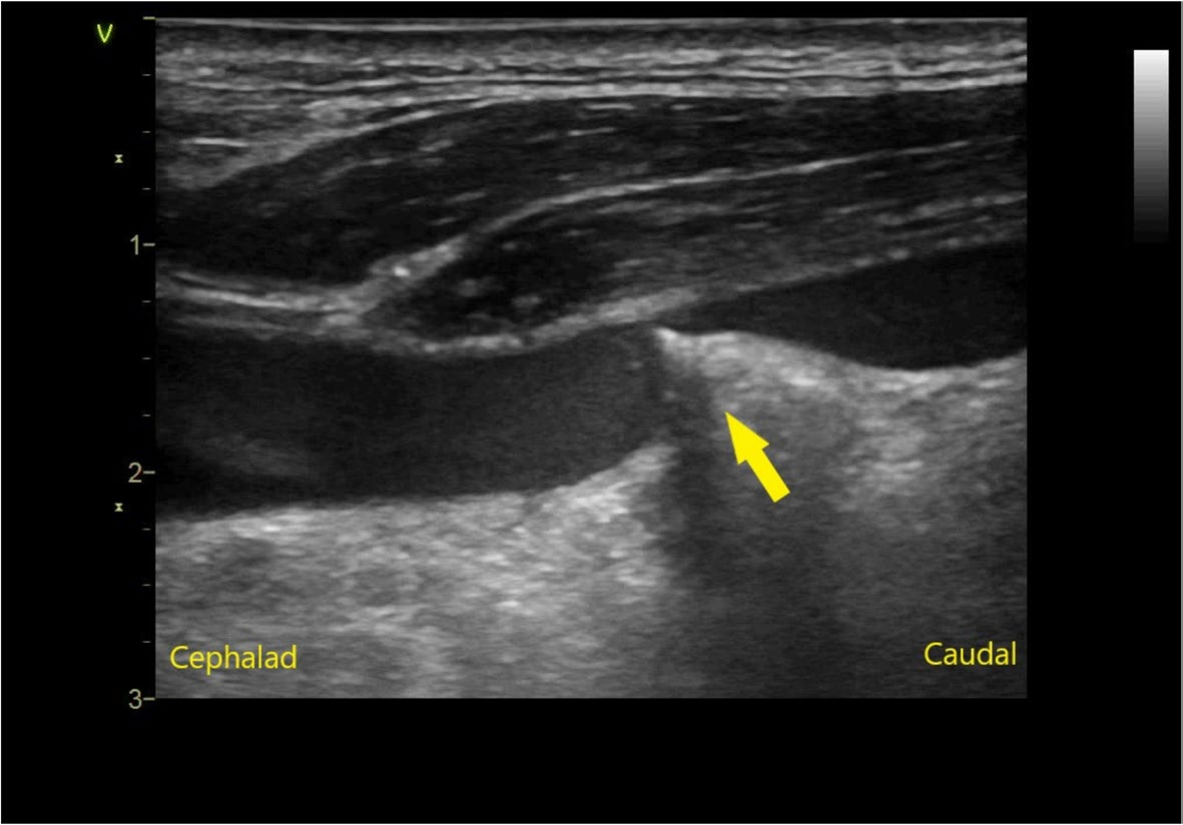
Ultrasound image of the left internal jugular vein in the longitudinal view. The posterior wall of the left internal jugular vein (arrow) is pulled ventrally by the guidewire which removal is attempted

**Fig. 2:**
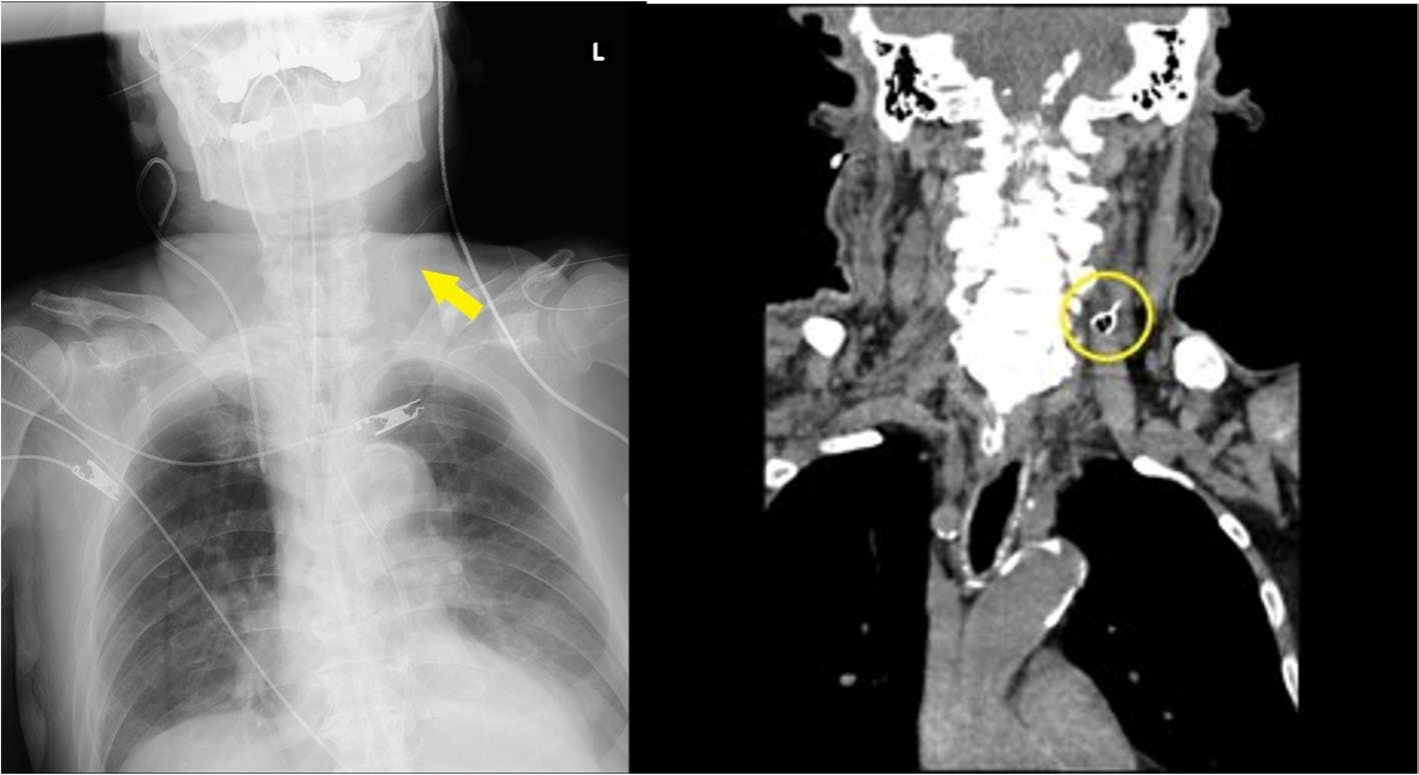
Chest radiograph (left) in the anteroposterior view, showing the guidewire looped in the neck (yellow arrow), and computed tomography of the neck (right), showing the tangled guidewire (yellow circle) near the left internal jugular vein

Using an out-of-plane, real-time US-guided technique, puncture of the left IJV was performed. After confirming blood aspiration, the guidewire was inserted up to the 20-cm mark through the Y-valve needle without resistance. After the needle was removed, ultrasound was performed to confirm the position of the guidewire. This demonstrated that the guidewire had penetrated the posterior wall of the vessel. The guidewire misplacement was promptly diagnosed by attending anesthesiologist. Although removal of the guidewire was attempted, there was a significant resistance during withdrawal, and further attempts at wire removal were not deemed safe. Ultrasound revealed that the posterior wall of the vessel moved toward the ventral side when an attempt was made to pull out the guidewire (Fig. 1). Chest radiograph and CT of the neck *demonstrated* that the guidewire had penetrated the posterior wall of the IJV and terminated in the left anterior scalene muscle, forming a knot (Fig. 2). A multi-disciplinary meeting of interventional radiologists, cardiac surgeons, anesthesiologists, intensive care physicians, and neurosurgeons was held, and it was decided that the guidewire would be removed by the interventional radiologist under fluoroscopy in the hybrid operating room. Surgical guidewire removal by the cardiac surgery team was planned as back-up.

**Fig. 3:**
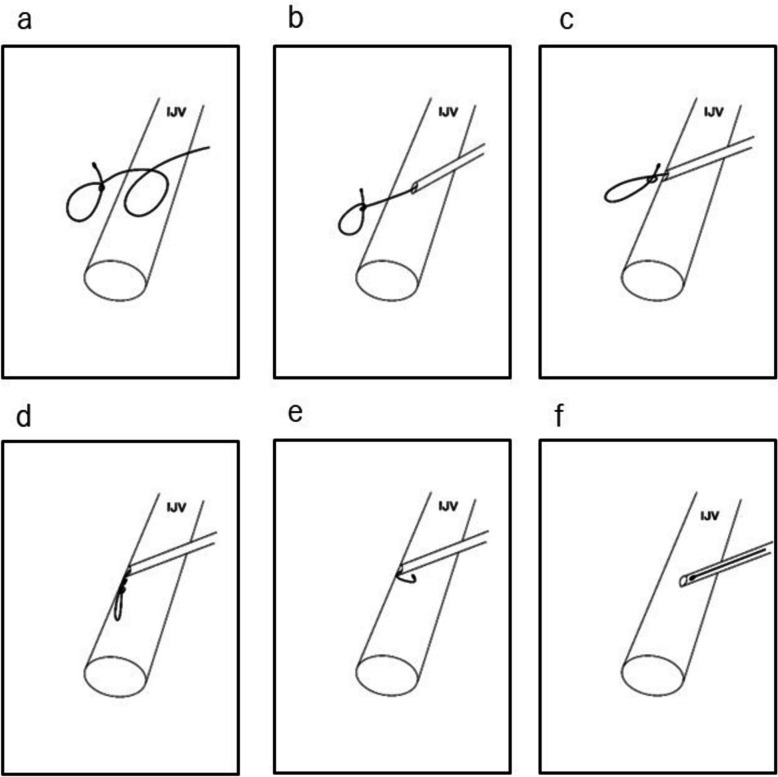
Frame-by-frame illustration of guidewire removal. **a** The guidewire, which had perforated the internal jugular vein, formed a knot as it was advanced into a confined space. **b** Insertion of a short sheath alleviated the slack in the guidewire within the vessel. **c** The short sheath was advanced to the posterior wall of the internal jugular vein, which helped stabilize the posterior wall. **d** Further manipulation of the guidewire allowed the knot to unravel due to the guidewire’s natural tendency to return to a straight configuration. **e** The guidewire was retrieved into the sheath. **f** The guidewire, along with the sheath, was removed

During guidewire removal, the patient was under general anesthesia, maintained with propofol, fentanyl, remifentanil, and rocuronium. Norepinephrine was administered to support blood pressure. A 4-French sheath was passed over the guidewire. Fluoroscopy showed that the knot was mobile using a push–pull technique. The sheath was advanced over the wire through the posterior wall of the IJ vessel. The knot was pulled into the sheath and untied (Fig. 3, supplemental video files). The guidewire was removed with the sheath. Compression hemostasis was applied for 20 min, and the absence of vascular injury or hematoma was confirmed using ultrasound before the patient was discharged from the operation room. Subsequent US imaging showed no evidence of vascular injury or hematoma formation. The time from the start of CVC placement to the end of guidewire was about 6 h.

The next CVC placement was performed via the right subclavian vein using the thin-walled needle technique, and the procedure was carried out by a PGY-6 physician.

## Discussion

CVC placement into the IJV is an invasive procedure that can result in mechanical complications, including arterial puncture, hematoma, and pneumothorax, occurring in 6.3–11.8% of cases [4]. Complications related to the guidewire placement are rare but also potentially serious [5]. Looping, kinking, or knotting of the guidewire may occur during CVC placement. Guidewire knotting during CVC placement was reported by Majek in 1975[6]. More recently, knotting of the guidewire during CVC placement in the subclavian vein was reported by Yong in 2013 [7] and Sekiguchi in 2022 [8]. Knotting of the guidewire has also been reported in other procedures, including cardiac catheterization [9], peripherally inserted central catheter placement [10], and biliary drainage [11]. Although the misplaced and knotted guidewire can be removed surgically, these techniques require invasive approaches [8, 12, 13]. Previous reports have shown that a 5-French sheath can be useful for removal of the knots in the guidewire [7, 14]. We report a novel approach using sheath placement under fluoroscopy. In this case, the guidewire knot was successfully removed under fluoroscopy using a push–pull technique in a hybrid operating room thereby avoiding a more invasive surgical procedure.

In our case, resistance to removal of the guidewire prompted the immediate performance of ultrasound, standard radiographic imaging, and CT imaging. These techniques led to a rapid diagnosis of the problem and precluded further attempts at simple manual removal of the knotted wire which may have resulted in vessel trauma or shearing of the wire within the patient. The diagnosis of the problem enabled consultation with other departments at an early stage and safe removal of the guidewire with minimal invasiveness in the hybrid operating room. The use of fluoroscopy provided invaluable in obtaining a comprehensive view of the wire and the complication that had occurred.

This complication may be avoided by using the over-the-needle catheter technique, which may be preferred over puncturing the vessel with a thin-wall needle. Lee et al. reported that the over-the-needle catheter technique had a higher first attempt success rate of needle and guidewire insertion (87.3% vs 77.3%, *p* = 0.037) and fewer puncture attempts (1.14 ± 0.4 vs 1.3 ± 0.6, *p* = 0.026) than puncture with a thin-wall during internal jugular vein catheterization; however, there was no difference in complications between the two groups [15]. Similar findings have been reported in neonates [16]. However, Kim et al. have shown that the over-the-needle technique increased the incidence of catheterization-related complications and decreased the first-time success rates when compared to the thin-wall needle technique[17]. In our case, the guidewire knot was believed to have formed as a result of the needle penetrating the posterior wall of the IJV during the insertion of the guidewire. It is possible that this complication would be less likely to occur with an over-the-needle catheter technique. In addition, it has been reported that the incidence of mechanical complications is reduced by 50% when the catheter is inserted by a clinically experienced physician [4]. Thus, in cases where catheter insertion is expected to be difficult, a clinically experienced physician should perform the insertion.

In conclusion, we present a very rare complication of a guidewire penetrating the posterior wall of the vessel and forming a knot. The case illustrates that it is imperative to adhere the fundamental principle of not extracting the guidewire by force when encountering resistance. The use of US, CT, and fluoroscopy was helpful for diagnosis, while the hybrid operating room proved invaluable for the removal procedure. Prospective trials are needed to further define whether needle placement technique (over-the-needle catheter vs. direct needle puncture) is beneficial in avoiding this complication. This anecdotal experience may be useful as the current clinical guidelines do not address the diagnosis and management of CVC-associated trauma or injury, with the exception of cases involving carotid arterial injury [3].

## Supplementary Information

Supplementary Material 1. Supplemental video file: Fluoroscopic imaging of the guidewire. A short sheath was inserted and then using a push–pull technique, the guidewire was unknotted and removed.

## Data Availability

Available from the corresponding author.

## Abbreviations

US: Ultrasound
CVC: Central venous catheter
CT: Computed tomography
IJV: Internal jugular vein
PGY: Post-graduate year
